# Quantitative assessment of glioblastoma phenotypes *in vitro* establishes cell migration as a robust readout of Crk and CrkL activity

**DOI:** 10.1016/j.jbc.2021.100390

**Published:** 2021-02-06

**Authors:** Taeju Park, Neka Large, Tom Curran

**Affiliations:** 1 Children's Mercy Research Institute, Children's Mercy Kansas City, Kansas City, Missouri, USA; 2 Department of Pediatrics, University of Missouri-Kansas City School of Medicine, Kansas City, Missouri, USA

**Keywords:** Crk, CrkL, glioblastoma, gene knockdown, cell migration, cell proliferation, cell invasion, cell adhesion, CIM-Plate, cell invasion and migration-plate, Crk, CT10 regulator of kinase, CrkL, Crk-like, DPT, day posttransfection, GBM, glioblastoma, NT1, nontargeting siRNA 1, *synGFP*, synthetic green fluorescent protein mRNA, *synRNA*, synthetic mRNA

## Abstract

The expression levels of CT10 regulator of kinase (Crk) and Crk-like (CrkL) are elevated in many human cancers, including glioblastoma (GBM), and are believed to contribute to poor prognosis. Although Crk and CrkL have been proposed as therapeutic targets in these tumors, the lack of a reliable, quantitative assay to measure Crk and CrkL activity has hindered development of inhibitors. Here, we knocked down Crk, CrkL, or both using siRNAs in a human GBM cell line, U-118MG, to determine the respective, quantitative contributions of Crk and CrkL to cellular phenotypes. The combined use of specific and potent Crk and CrkL siRNAs induced effective knockdown of CrkII, CrkI, and CrkL. Whereas Crk knockdown did not affect cell morphology, proliferation, adhesion, or invasion, CrkL knockdown caused shrinkage of cells and inhibition of cell proliferation, adhesion, and invasion. Crk/CrkL double knockdown resulted in more pronounced morphological alterations and more robust inhibition of proliferation, adhesion, and invasion. Furthermore, Crk/CrkL double knockdown completely blocked cell migration, and this effect was rescued by transient overexpression of CrkL but not of Crk. Quantification of protein levels indicated that CrkL is expressed more abundantly than CrkII and CrkI in U-118MG cells. These results demonstrate both the predominant role of CrkL and the essential overlapping functions of Crk and CrkL in U-118MG cells. Furthermore, our study indicates that migration of U-118MG cells depends entirely on Crk and CrkL. Thus, impedance-based, real-time measurement of tumor cell migration represents a robust assay for monitoring Crk and CrkL activities.

Glioblastoma (GBM) is the most common aggressive primary malignant brain tumor, with an average of 15 months of survival after diagnosis (https://www.cancer.gov/about-nci/organization/ccg/research/structural-genomics/tcga/studied-cancers/glioblastoma) (1). Approximately 13,310 new cases of GBM were estimated in the United States in 2019, according to the American Brain Tumor Association (https://www.abta.org/wp-content/uploads/2018/12/glioblastoma-anaplastic-astrocytoma.pdf). Standard therapy for GBM is surgical removal of the tumor, followed by ionizing radiation and chemotherapy. The success of this approach depends primarily on how completely the tumor is surgically resected, in part because effective chemotherapy is not available. However, complete resection of GBM is challenging because the tumor is heterogeneous and highly infiltrative (2, 3, 4). Recent reports suggest that GBM cells are very motile, and degree of motility negatively correlates with survival of patients (5, 6). Therefore, it is necessary to develop effective chemotherapy to minimize infiltration of tumor cells into surrounding tissues (7). A combination of surgery, radiation, and chemotherapy that blocks GBM cell motility is required to improve prognosis.

CT10 regulator of kinase (Crk) and Crk-like (CrkL) are overexpressed in multiple human cancer types, including GBM (8, 9, 10), breast cancer (11, 12, 13), non-small cell lung cancer (14, 15, 16), gastric cancer (17, 18, 19), cervical cancer (20, 21), ovarian cancer (22, 23), endometrial carcinoma (24), oral squamous cell carcinoma (25), bladder cancer (26), rhabdomyosarcoma (27), thyroid cancer (28), pancreatic cancer (29), and colorectal cancer (30). Reduced expression of either Crk or CrkL, by RNA interference-mediated gene knockdown, inhibited cell adhesion, migration, invasion, proliferation, and *in vivo* tumor growth of many cancer cell lines such as GBM (31, 32), breast cancer (11, 12, 13), gastric cancer (17, 33), cervical cancer (34), ovarian cancer (22, 23, 35), prostate cancer (36, 37), bladder cancer (26), oral squamous cell carcinoma (25), synovial sarcoma (38), rhabdomyosarcoma (27), hepatocellular carcinoma (39), and head and neck squamous cell carcinoma (40) cells. In addition, ablation of Crk by CRISPR/Cas9 inhibited *in vivo* growth of breast cancer cells (41), and ablation of both Crk and CrkL by CRISPR/Cas9 inhibited colorectal cancer cell adhesion, migration, invasion, and proliferation (42). Furthermore, elevated expression of Crk or CrkL correlated with the poor prognosis in GBM (9), lung cancer (16, 43), gastric cancer (19), ovarian cancer (44), oral squamous cell carcinoma (25), pancreatic cancer (29), and colorectal cancer (30). Therefore, Crk and CrkL have been proposed as potential therapeutic targets in these cancers. Although it is well known that Crk and CrkL are structurally and functionally similar, the majority of studies address the role of either Crk or CrkL individually in knockdown or overexpression experiments in cancer cell models, in gene expression analyses of cancer tissues, and in outcome analyses of patient survival. Thus, the scientific literature has created an enigma; it appears that Crk and CrkL are important in several cancers, but it is unclear whether a single one of these genes is the culprit or both in combination. This uncertainty is compounded by the fact that most knockdown studies, targeting either Crk or CrkL alone, did not investigate the possibility of cross-regulation. Therefore, the siRNAs or shRNAs used in these studies may not have specifically inhibited either Crk or CrkL, because of their high level of sequence similarity, as they may have also affected expression of the paralog. Similarly, a study in which shRNAs were used to reduce expression of CrkII, CrkI, and CrkL simultaneously did not ascertain whether the phenotypes observed required knockdown of Crk and CrkL in combination or just one of the genes alone (12). Recently, Franke *et al.* (42), using individual and double knockout cells, demonstrated that Crk and CrkL both contribute to migration and invasion of colon cancer cells.

Previously, we reported that Crk and CrkL play essential overlapping roles in many biological processes, including neuronal migration, neuromuscular synapse formation, podocyte morphogenesis, T cell migration into sites of inflammation, lens fiber cell elongation, and natural killer cell expansion and differentiation during mouse cytomegalovirus infection (45, 46, 47, 48, 49, 50). In addition, we induced knockout of Crk and CrkL in cultured fibroblasts using the Cre-loxP recombination and demonstrated that Crk and CrkL play essential overlapping roles in cell structure, motility, and growth (51, 52). These studies indicate that a more systematic and comprehensive analysis is required to untwine the individual or collective contributions of Crk and CrkL to tumor cell biology. This concern prompted us to compare single and double knockdown of Crk and CrkL in a GBM cell line using a systematic and quantitative approach to address effects on cell adhesion, migration, invasion, and proliferation. Our results demonstrate that CrkL, which is more abundantly expressed than CrkII and CrkI, plays a more prominent role than Crk and that Crk and CrkL play essential overlapping roles in cell adhesion, migration, invasion, and proliferation in the GBM cell line investigated. Our study supports the use of impedance-based real-time measurement of cell migration as a quantitative assay for monitoring activities of Crk and CrkL.

## Results

### Optimization of GBM cell transfection

Previously, we achieved a rapid and efficient transfection of mouse embryonic fibroblasts with synthetic mRNA (*synRNA*) using the Neon electroporation system (52). We applied this method to transfect the GBM cell line U-118MG. When U-118MG cells were electroporated with synthetic green fluorescent protein mRNA (*synGFP*), many cells showed strong green fluorescence (Fig. S1*A*). The viability of cells was indirectly assessed by the initial measurement of the WST-1 signal. Upon electroporation, viabilities were not different among cells electroporated without and with *synGFP* (Fig. S1*B*; 208 ± 14 for none and 199 ± 8 for *synGFP*). The growth rates over 2 days were also not affected significantly by the electroporation of cells with *synGFP* (Fig. S1*C*). The results suggest that RNA can be efficiently introduced to U-118MG cells by electroporation using the Neon system without any noticeable nonspecific changes in cell viability and proliferation.

### Testing of Crk siRNAs

**Figure 1:**
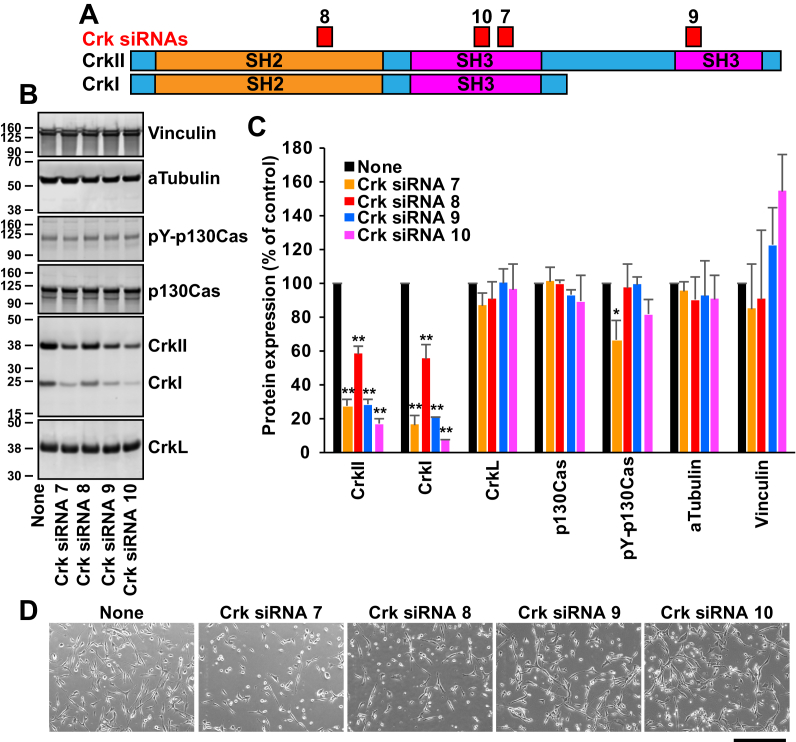
**Electroporation of U-118MG cells with Crk siRNAs.***A*, a schematic diagram of the target areas of Crk siRNAs. *B*, U-118MG cells were electroporated with Crk siRNAs (40 pmol in 10 μl cell suspension), and total cell lysates were prepared at 3 days posttransfection (DPT) for the Western blot analyses. Protein levels upon siRNA transfection were compared with those upon electroporation without siRNAs. Alpha-tubulin and vinculin levels were measured as controls. *C*, protein bands were quantified using the Odyssey system, and their mean ± standard deviation (SD) values are shown. ∗*p* < 0.05, ∗∗*p* < 0.01, compared with the control (none). *D*, phase-contrast images of live cells were taken at 3 DPT using the EVOS system. Representative images are shown. Scale bar: 400 μm. Crk, CT10 regulator of kinase.

To find siRNAs that induce efficient and specific knockdown of Crk, we transfected U-118MG cells with four different siRNAs targeting the SH2 or SH3 domains of the human *CRK* gene (Fig. 1*A*). Then, we examined the level of Crk and CrkL proteins produced at 3 days posttransfection (DPT). Crk siRNAs 7, 9, and 10 reduced expression levels of CrkII and CrkI by more than 70% without affecting CrkL expression (Fig. 1, *B* and *C*). Crk siRNA 10 was the most effective at reducing CrkII and CrkI levels (83.2 ± 2.9% and 92.6 ± 0.2%, respectively). On the other hand, Crk siRNA 8 reduced the CrkI and CrkII expression by 42.0 ± 4.4% and 44.8 ± 8.8%, respectively. Although Crk siRNAs 7 and 9 reduced CrkI and CrkII expression by similar amounts, only Crk siRNA 7 decreased the phospho-p130Cas level significantly, suggesting that the inhibition of p130Cas phosphorylation by Crk siRNA 7 might not be caused by the reduction of CrkII and CrkI. We also examined the effect of gene knockdown on cell morphology. Whereas Crk siRNAs 7 and 8 caused visible morphological alterations, Crk siRNAs 9 and 10 did not cause apparent morphological alterations (Fig. 1*D*), suggesting that the morphological alterations caused by Crk siRNAs 7 and 8 may not be mediated by the reduction in the CrkI and CrkII levels. Interestingly, CrkI expression was decreased by Crk siRNA 9, although CrkI lacks the target site for the siRNA. It is unclear whether there is any feedback regulation of CrkI and CrkII at the mRNA and protein levels. We selected Crk siRNA 10 for future studies because it was the most potent siRNA among those tested without affecting the phospho-p130Cas level and the cell morphology.

### Testing of CrkL siRNAs

**Figure 2:**
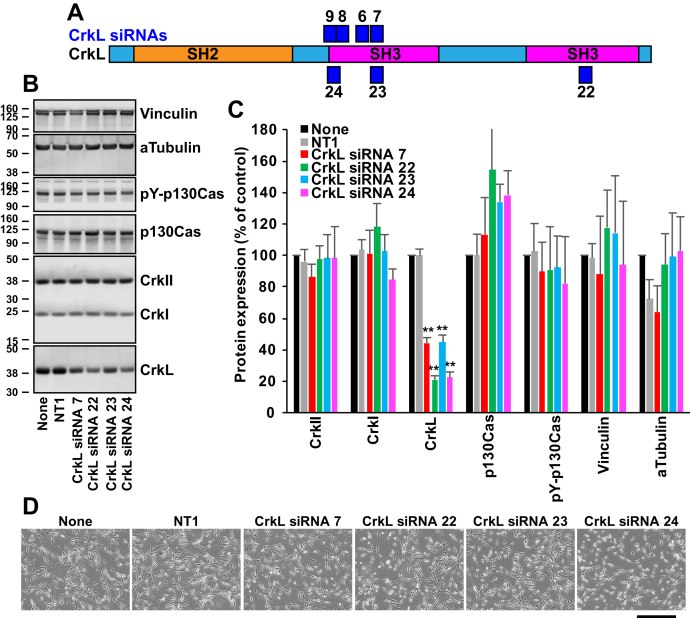
**Electroporation of U-118MG cells with additional CrkL siRNAs.***A*, a schematic diagram of the target areas of CrkL siRNAs. *B*, U-118MG cells were electroporated with CrkL siRNAs (40 pmol in 10 μl cell suspension), and total cell lysates were prepared at 4 DPT for Western blot analyses. Protein levels upon siRNA transfection were compared with those upon electroporation without siRNAs (none) or with nontargeting siRNA 1 (NT1). Alpha-tubulin and vinculin levels were measured as controls. *C*, protein bands were quantified using the Odyssey system, and their mean ± SD values are shown. ∗∗*p* < 0.01, compared with the control (NT1). *D*, phase-contrast images of live cells were taken using the EVOS system at 4 DPT. Representative images are shown. Scale bar: 400 μm. Crk, CT10 regulator of kinase; CrkL, Crk-like; DPT, days posttransfection.

We took a similar approach to find siRNAs that induce specific knockdown of CrkL. U-118MG cells were transfected with four different siRNAs targeting the N-terminal SH3 domain of the human *CRKL* gene (Fig. S2*A*), and protein levels were examined at 3 DPT. CrkL siRNA 7 was the most effective, reducing CrkL expression by 71.3 ± 0.9%. However, it also reduced the levels of CrkI and phospho-p130Cas significantly (Fig. S2, *B* and *C*). In addition, CrkL siRNA 7 appeared to induce modest morphological alterations (Fig. S2*D*). Therefore, we tested additional siRNAs to find more potent and specific CrkL siRNAs (Fig. 2*A*). We used nontargeting siRNA 1 (NT1) as a negative control. Whereas CrkL siRNA 23 showed similar effects to CrkL siRNA 7, both CrkL siRNAs, 22 and 24, showed more robust reductions in CrkL expression (by 79 ± 2.4% and 77.5 ± 3.3%, respectively) without affecting CrkII, CrkI, and other proteins tested (Fig. 2, *B* and *C*). While CrkL siRNA 22 did not cause morphological alterations, CrkL siRNA 24 slightly affected cell morphology (Fig. 2*D*). The results suggest that CrkL siRNA 22 may be the most specific and potent siRNA among those tested. Notably, the target sequence for both CrkL siRNAs 7 and 23 was identical, although they were purchased from different companies, which explains their similar effects. These results demonstrate the reproducibility of our experiments.

Next, we tested different concentrations of Crk and CrkL siRNAs to identify the optimal amounts of siRNAs to produce potent and specific gene knockdown. The knockdown effect of Crk siRNA 10 on the CrkII and CrkI levels increased as the siRNA concentration increased and reached the maximum at around 40 pmol (85.4 ± 4.1% for CrkII and 92.5 ± 2.6% for CrkI) (Fig. S3, *A* and *B*). The knockdown effect caused by Crk siRNA 10 appears to be very specific because, at levels up to 80 pmol, it did not affect the expression of CrkL or other proteins tested. Crk siRNA 10 did not induce any apparent morphological alterations (Fig. S3*C*). In contrast, the knockdown of CrkL by siRNA 22 seemed to continually increase in a dose-dependent manner across the range tested. Therefore, we examined whether the combined transfection of cells with CrkL siRNAs 22 and 24 induces more potent knockdown effects. When cells were transfected with CrkL siRNAs individually, both CrkL siRNAs 22 and 24 exhibited similar reductions in CrkL expression at 40 and 80 pmol (Fig. S4, *A* and *B*). When cells were transfected with both CrkL siRNAs 22 and 24 in combination, the inhibitory effect at a total of 80 pmol (82.8 ± 1.4%) was slightly higher than at a total of 40 pmol (79.1 ± 1.5%). The inhibitory effect induced by the combined transfection was a little higher than that produced by transfections with the individual siRNAs (76.5 ± 2.2% and 79.2 ± 1.9% for CrkL siRNAs 22 and 24, respectively) even though the total amount of siRNAs was 80 pmol in both cases. The morphological alterations associated with the combined transfection with CrkL siRNAs 22 and 24 appeared to be similar to CrkL siRNA 22 alone at a total of 80 pmol (Fig. S4*C*). Therefore, we chose to use both CrkL siRNAs 22 and 24 (40 pmol each) to induce CrkL knockdown.

### Effects of Crk and CrkL knockdown on cell proliferation and morphology

**Figure 3:**
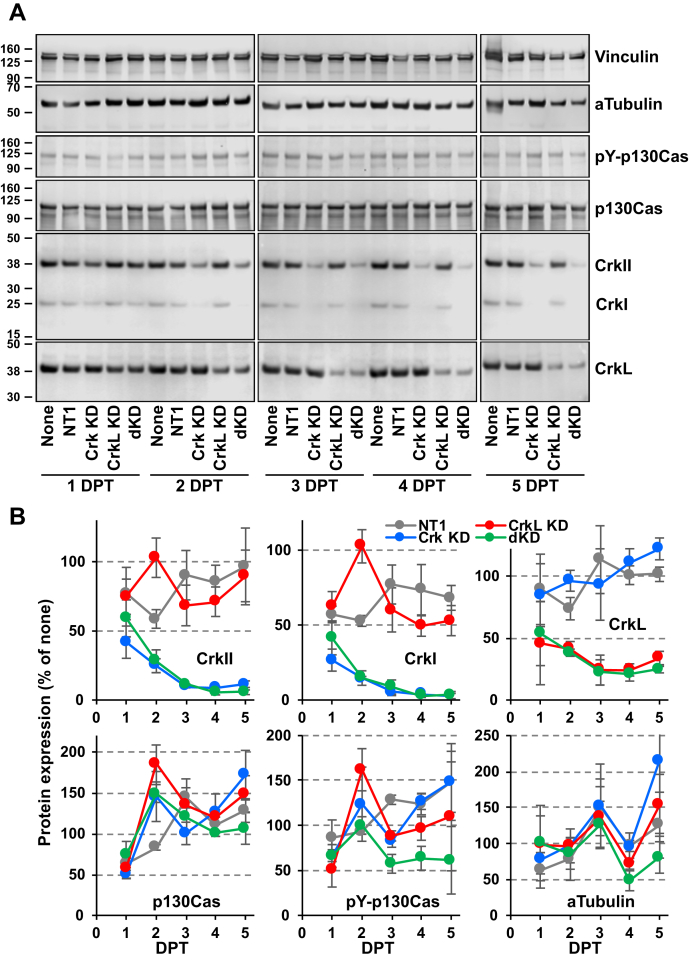
**Time courses of Crk and CrkL knockdown.***A*, U-118MG cells were electroporated with NT1 (80 pmol), Crk siRNA 10 (40 pmol), CrkL siRNAs 22 (20 pmol) plus 24 (20 pmol), or with Crk and CrkL siRNAs together, and total cell lysates were prepared at the indicated DPT for Western blot analyses. Protein levels upon siRNA transfection were compared with those upon electroporation without siRNAs (none) at the indicated time point. Alpha-tubulin and vinculin levels were measured as controls. *B*, protein bands were quantified using the Odyssey system, calculated as percentages of the control cells (none), and their mean ± SD values are shown. Two independent experiments were carried out, and the results were reproducible. Crk, CT10 regulator of kinase; CrkL, Crk-like; DPT, days posttransfection; NT1, nontargeting siRNA 1.

**Figure 4:**
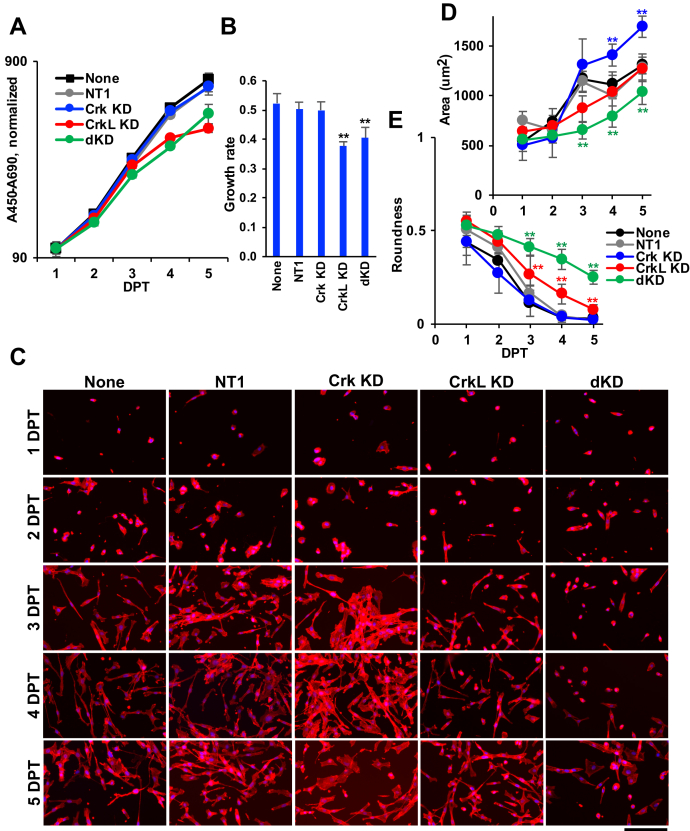
**Effects of Crk and CrkL knockdown on cell proliferation and morphology.***A*, U-118MG cells were electroporated with NT1 (80 pmol), Crk siRNA 10 (40 pmol), CrkL siRNAs 22 (20 pmol) plus 24 (20 pmol), or with Crk and CrkL siRNAs together, and cell proliferation was quantitatively measured using WST-1. The A_450_–A_690_ values are presented in the logarithmic scales. *B*, exponential trendlines for the WST-1 assay graphs were drawn, and their slopes, the coefficients of *x*, are presented as the rates for exponential cell growth. ∗∗*p* < 0.01, compared with NT1. *C*–*E*, after cells were transfected with Crk and CrkL siRNAs, the cells were fixed at the indicated DPT and stained with phalloidin and DAPI to visualize the cytoplasm and the nucleus, respectively. Representative images are shown (*C*). Scale bar: 400 μm. Cell surface areas (*D*) and roundness (*E*) of phalloidin-stained objects were calculated according to the Experimental procedures, and their mean ± SD values are shown. ∗∗*p* < 0.01, compared with both none and NT1 at the same DPT. Two independent experiments were carried out, and the results were reproducible. Crk, CT10 regulator of kinase; CrkL, Crk-like; DPT, days posttransfection; NT1, nontargeting siRNA 1.

We transfected U-118MG cells with Crk siRNA 10 and CrkL siRNAs 22 and 24 and carried out time course experiments to examine when single and double knockdown of Crk and CrkL reached peak effects. As shown in Figure 3, *A* and *B*, the CrkII and CrkI levels, upon Crk knockdown, continued to decrease until 4 DPT, at which point the inhibitory effects reached a maximum (91.4 ± 1.4% for CrkII and 96.3 ± 0.4% for CrkI). Upon CrkL knockdown, the CrkL protein level decreased until 3 DPT, reaching maximal inhibition at 3 and 4 DPT (by 75.8 ± 12.0% and 75.7 ± 4.9%, respectively). Upon the double knockdown of both Crk and CrkL, protein levels of CrkII, CrkI, and CrkL were the lowest at 4 DPT. The phospho-p130Cas level in cells undergoing double knockdown was significantly lower than the control levels at 3 and 4 DPT. Because fibroblast proliferation was blocked by ablation of Crk and CrkL using the Cre-loxP recombination (52), we examined cell proliferation under single and double knockdown conditions. Decreases in cell proliferation by CrkL knockdown and Crk/CrkL double knockdown became evident at 4 DPT (Fig. 4*A*), which is consistent with the prior observation that the maximum knockdown occurred at 4 DPT. When assessed by the growth rate, Crk knockdown did not affect proliferation of U-118MG cells, whereas CrkL knockdown inhibited cell proliferation by approximately 25%, compared with NT1 (Fig. 4*B*). Double knockdown of Crk and CrkL also exhibited a similar extent of inhibition to CrkL knockdown, suggesting that cell proliferation partially depends on CrkL.

Next, we analyzed cell morphology to determine whether knockdown of Crk and CrkL affected cell structure. A comparison of phase-contrast images of cells taken using EVOS suggested that cells looked different with CrkL knockdown and Crk/CrkL double knockdown at 3 to 5 DPT (Fig. S5). To analyze the cell morphology quantitatively, we stained the cells with fluorophore-conjugated phalloidin and calculated the cytoplasmic area and roundness. Images of phalloidin-stained cells suggested that cells looked sparse with Crk/CrkL double knockdown at 3 to 5 DPT, compared with the controls (none and NT1) (Fig. 4*C*). The measurement of cytoplasmic area indicated that cells gradually spread with an increase in cytoplasmic area as the day progressed after the transfection and plating. The cytoplasmic areas of the control cells (none and NT1) increased gradually from 1 DPT to 5 DPT (Fig. 4*D*). The gradual spreading of cells on culture dishes was moderately inhibited upon Crk/CrkL double knockdown at 3 to 5 DPT (1035 ± 123 μm^2^, compared with 1281 ± 139 μm^2^ for NT1 at 5 DPT) (Fig. 4*D*). The inhibition of cell spreading or the decrease in cell surface area in the absence of Crk and CrkL is consistent with our previous reports with fibroblasts (51, 52). On the other hand, Crk knockdown cells exhibited enhanced spreading at 4 and 5 DPT, but the increases were not significant when the experiment was repeated (data not shown). Lack of correlation between cell number and cell surface area in a field of view for each sample at 5 DPT suggests that the altered cell morphology may not be the outcome of cell density changes (data not shown).

We also investigated the roundness of cells. Control cells became less rounded as the days progressed after transfection and plating (Fig. 4*E*). These decreases in roundness were consistent with increases in the cytoplasmic area as cells continued to spread (Fig. 4*D*). The decrease in roundness significantly subsided upon CrkL knockdown and Crk/CrkL double knockdown (Fig. 4*E*). In contrast, Crk knockdown did not affect the change in roundness. The morphological alterations, assessed by the cytoplasmic area and the roundness, were more significant with Crk/CrkL double knockdown than with CrkL knockdown (Fig. 4, *D* and *E*). In contrast, the nuclear area and roundness of control cells did not change after transfection and plating and were not affected by knockdown of Crk and CrkL (Fig. S6, *A* and *B*). The results suggest that reorganization of the cell cytoskeleton is defective in the absence of Crk and CrkL, with CrkL playing a predominant role. The results also emphasize that quantitative analyses are critical in comparing single and double knockdown effects of Crk and CrkL to obtain a clear view of their essential overlapping functions.

We examined distribution of Crk and CrkL expression in the cell population upon single and double knockdown using flow cytometry analyses. Cells with single and double knockdown were fixed and stained with anti-Crk and anti-CrkL antibodies that were used for the Western blot analyses. To make any changes caused by the knockdown easily interpretable, we divided the fluorescence signal into 11 bins and conducted a bin analysis. As shown in Figure S7, *A* and *B*, the signal detected by the anti-Crk antibody shifted to the left upon Crk knockdown and Crk/CrkL double knockdown, suggesting that the signal is specific to Crk. There was a range of Crk expression in control cells, which moved to the left upon Crk knockdown. However, a majority of Crk knockdown cells exhibited still higher signals than the control cells, suggesting that most cells had a substantial, but not complete, knockdown. On the other hand, the signal detected by the anti-CrkL antibody did not shift upon CrkL knockdown and Crk/CrkL double knockdown, suggesting that the signal is not specific to CrkL (Fig. S7, *A* and *C*).

### Effects of Crk/CrkL knockdown after harvesting cells and re-plating at 3 DPT

**Figure 5:**
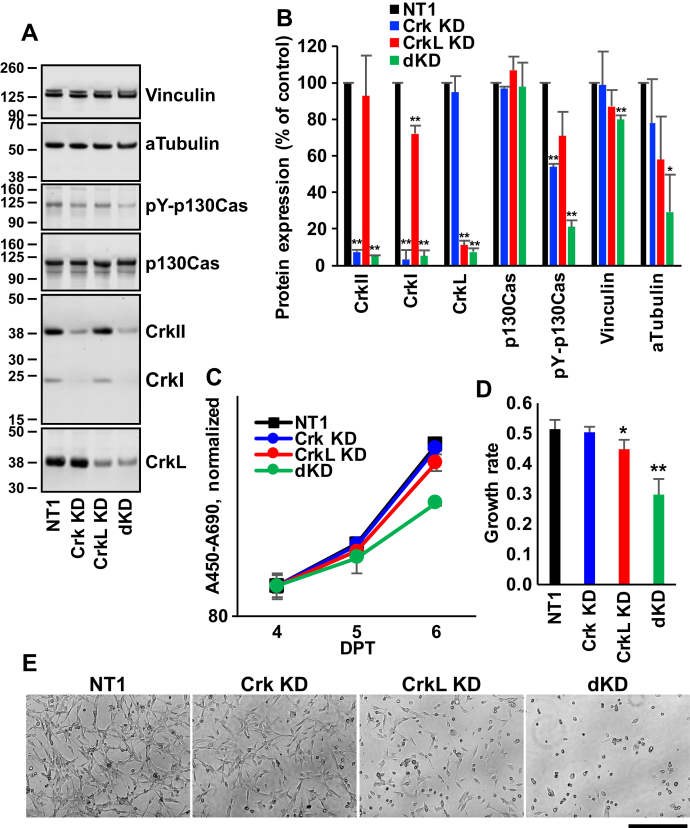
**Effects of Crk and CrkL knockdown after re-plating at 3 DPT.***A*, U-118MG cells were electroporated with NT1 (80 pmol), Crk siRNA 10 (40 pmol), CrkL siRNAs 22 (20 pmol) plus 24 (20 pmol), or with Crk and CrkL siRNAs together. The cells were harvested and re-plated at 3 DPT, and total cell lysates were prepared at 4 DPT for Western blot analyses. Protein levels upon siRNA transfection were compared with NT1. Alpha-tubulin and vinculin levels were measured as controls. *B*, protein bands were quantified using the Odyssey system, calculated as percentages of the control (NT1), and their mean ± SD values are shown. ∗*p* < 0.05, ∗∗*p* < 0.01, compared with NT1. *C*, proliferation of U-118MG cells electroporated with Crk and CrkL siRNAs after re-plating at 3 DPT was quantitatively measured using WST-1. The A_450_–A_690_ values are presented in the logarithmic scales. *D*, exponential trendlines for the WST-1 assay graphs were drawn and their slopes, the coefficients of *x*, are presented as the rates for exponential cell growth. *E*, after cells were transfected with Crk and CrkL siRNAs followed by re-plating at 3 DPT, phase-contrast images of live cells were taken at 4 DPT using the EVOS system. Representative images are shown. Scale bar: 400 μm. Three independent experiments were carried out, and the results were reproducible. Crk, CT10 regulator of kinase; CrkL, Crk-like; DPT, days posttransfection; NT1, nontargeting siRNA 1.

Crk and CrkL have been reported to contribute to adhesion, migration, and invasion of GBM cell lines (8, 9, 10, 31, 32). To quantitatively analyze the effects of single and double knockdowns of Crk and CrkL on cell adhesion, migration, and invasion, we recently established impedance-based real-time cell assays using the xCELLigence system (Agilent) (53). The system provided real-time, quantitative, and comprehensive analyses of cancer cell behavior (9, 10, 53). However, unlike the other assays that we used, real-time cell analysis requires suspended cells. Because gene knockdown of Crk and CrkL takes 3 to 4 days to reach maximum after transfection, we harvested cells at 3 DPT and carried out impedance-based cell analyses as well as other experiments. Because we carried out the previous experiments without harvesting cells after transfection, we repeated the previous experiments under modified culture conditions. First, we examined the protein levels at 4 DPT (1 day after harvesting and re-plating cells) to compare the results between the two experimental conditions. As shown in Figure 5, *A* and *B*, Crk knockdown decreased the CrkII and CrkI expression to 7.6 ± 1.1% and 3.0 ± 5.6% at 4 DPT (compared with 8.6 ± 1.4% and 3.7 ± 0.4%, respectively in Fig. 3*B*). CrkL knockdown decreased CrkL expression to 11.3 ± 2.5% (compared with 24.3 ± 4.9% in Fig. 3*B*). In addition, alpha-tubulin levels observed in the Crk/CrkL double knockdown conditions were similar in previous (Fig. 3*B*) and current (Fig. 5*B*) experiments. These findings suggest that reductions in CrkII, CrkI, and CrkL protein levels are comparable between the two experiments.

Phospho-p130Cas levels were significantly lower in cells undergoing Crk knockdown and Crk/CrkL double knockdown at 4 DPT (Fig. 5*B*). In the previous experiment, phospho-p130Cas was significantly lower at 4 DPT only with Crk/CrkL double knockdown (Fig. 3*B*). Although it is unclear how the change in cell culture influenced this difference, cell adhesion and spreading after re-plating under the Crk and CrkL knockdown condition may have rendered the phospho-p130Cas level more vulnerable. p130Cas is known to be phosphorylated upon fibroblast adhesion (54), and cell spreading was delayed in the absence of p130Cas (55). In contrast, cells adhered and spread before complete suppression of *CRK*/*CRKL* gene expression in the previous experiment. Nevertheless, Crk/CrkL double knockdown resulted in a decrease in p130Cas phosphorylation under both conditions.

Next, we compared the effects of Crk and CrkL knockdown on cell proliferation in the two culture conditions. Similar to the previous experiment (Fig. 4, *A* and *B*), Crk knockdown did not affect cell proliferation (Fig. 5, *C* and *D*). In addition, inhibition of cell proliferation following CrkL knockdown was consistent. On the other hand, Crk/CrkL double knockdown induced more potent inhibition in cell proliferation (42%, compared with 19% in Fig. 4*B*). This difference suggests that we observed a more potent inhibitory effect caused by double knockdown because we started the cell proliferation assay after 3 DPT, a time when Crk and CrkL were fully suppressed. The current experiment shows modest inhibition of cell proliferation by CrkL knockdown and a potent inhibition by Crk/CrkL double knockdown. We also compared the effects of Crk and CrkL knockdown on cell morphology. Phase-contrast imaging at 4 DPT (1 day after re-plating) revealed morphological alterations induced by CrkL knockdown and by Crk/CrkL double knockdown (Fig. 5*E*). The morphological alterations were more pronounced than those observed in the previous experiment in the absence of re-plating (Fig. S5), suggesting that cell adhesion and spreading after re-plating may have enhanced the knockdown effects. Both the previous and the current experiments consistently show modest morphological alterations resulting from CrkL knockdown and more significant morphological alterations associated with Crk/CrkL double knockdown. Comparison of the results obtained from the two culture conditions indicate that harvesting and re-plating cells at 3 DPT is a reasonable and effective approach to investigate the effects of knockdown. This approach allowed us to observe knockdown effects more clearly by avoiding the 1 to 3 DPT period when the level of gene knockdown was increasing.

### Effects of Crk/CrkL knockdown on cell adhesion, migration, and invasion

**Figure 6:**
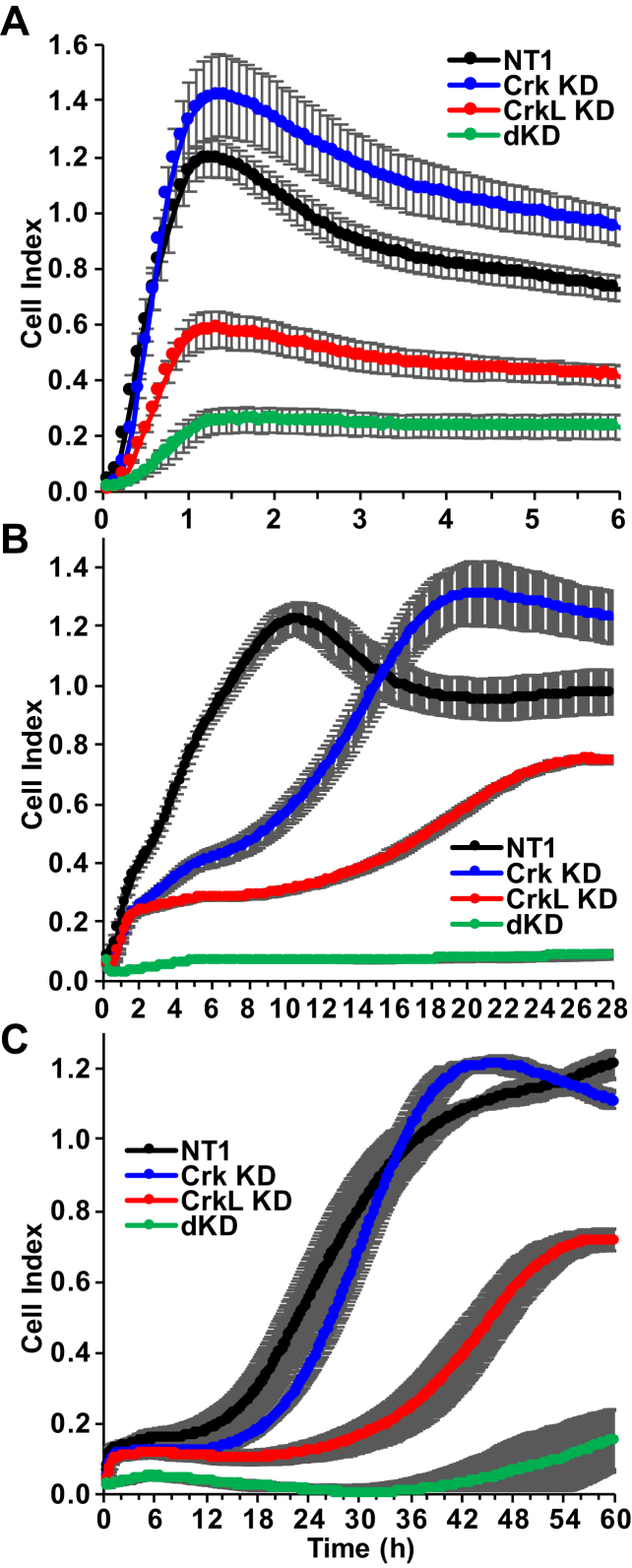
**Effects of Crk and CrkL knockdown on cell adhesion, migration, and invasion.** U-118MG cells were electroporated with NT1 (80 pmol), Crk siRNA 10 (40 pmol), CrkL siRNAs 22 (20 pmol) plus 24 (20 pmol), or with Crk and CrkL siRNAs together. At 3 DPT, the cells were harvested and plated onto an E-Plate 16, and two CIM-Plates without and with Matrigel coating for cell adhesion (*A*), migration (*B*), and invasion (*C*) using the xCELLigence system according to the Experimental procedures. Cell index values were obtained from four wells for each sample, and their mean ± SD values are shown. Three independent experiments were carried out, and the results were reproducible. CIM, cell invasion and migration; Crk, CT10 regulator of kinase; CrkL, Crk-like; DPT, days posttransfection; NT1, nontargeting siRNA 1.

We induced knockdowns and harvested cells at 3 DPT for impedance-based real-time cell analyses using xCELLigence. First, we tested the effects of Crk and CrkL knockdown on adhesion of GBM cells to fibronectin-coated plates. Adhesion of control cells to fibronectin following NT1 transfection took place quickly and reached a maximum (1.19 ± 0.07) after 1 h (Fig. 6*A*). In the case of Crk knockdown, there was a modest increase in maximum adhesion (1.42 ± 0.15). On the other hand, CrkL knockdown alone resulted in decreased adhesion (0.58 ± 0.06). Crk/CrkL double knockdown further reduced cell adhesion (0.25 ± 0.04). Our results suggest that Crk does not seem to play a significant role in cell adhesion in the presence of CrkL. However, when CrkL levels are low, cells depend on Crk for GBM cell adhesion.

We also examined the effects of Crk and CrkL knockdown on GBM cell migration. The cell index increased as time passed, indicating that cells placed in the upper chamber with low serum migrated toward the lower chamber with high serum through the microporous membrane and deposited onto the impedance electrode on the underside of the upper chamber (53). The cell index for the control cells with NT1 transfection continually increased until it reached the maximum at approximately 11 h (Fig. 6*B*). Then, the cell index slowly decreased for several hours, suggesting that more cells, after the migration, detached from the electrode surface into the medium in the lower chamber than the cells that newly migrated through the membrane. Therefore, we focused on the maximal cell index and the time for cells to reach that maximal point for each condition. Cells with Crk knockdown migrated at slower rates than the control cells, reaching the maximum at about 20 h. However, maximal migrations were similar between control and Crk knockdown cells (1.23 ± 0.06 and 1.31 ± 0.11, respectively). On the other hand, cells with CrkL knockdown showed slower rates of migration, and maximal migration was significantly lower (0.75 ± 0.01 at 26 h). These results suggest that both Crk and CrkL contribute to cell migration, probably through different mechanisms. Furthermore, cells with Crk/CrkL double knockdown failed to migrate. The failure of migration by Crk/CrkL knockdown cells was much more severe than a projected delay in migration of CrkL knockdown cells, which implies that Crk and CrkL cooperate and play essential overlapping roles in GBM cell migration. The results are consistent with our previous observations (53), and they emphasize the importance of comparing single and double knockdown cell populations to uncover the overlapping functions of Crk and CrkL.

We also tested the effects of Crk and CrkL knockdown on the GBM cell invasion. Cell invasion was measured by coating the top of the microporous membrane with Matrigel. Unlike cell migration, there was an initial lag phase of about 10 h before the control cells started to invade the Matrigel and reach the impedance electrode on the underside of the upper chamber (Fig. 6*C*). After cells migrated through the Matrigel for about 24 h, cell invasion was reduced. Cells with Crk knockdown showed a similar pattern of invasion, reaching maximum (1.21 ± 0.02) at 43 h. Cells with CrkL knockdown had a more prolonged lag phase of approximately 22 h before they migrated through the Matrigel, continuing for about 34 h with invasion reaching maximum values at 57 h. Crk/CrkL double knockdown further prolonged the lag phase to about 33 h, and invasion at 60 h reached lower values (0.16 ± 0.08) than that observed following Crk knockdown (1.11 ± 0.02) or CrkL knockdown (0.72 ± 0.03). It is unclear whether the slight increase in cell invasion after 42 h for the Crk/CrkL double knockdown cells reflects delayed cell invasion or invasion of newly proliferating cells. These results demonstrate both a prominent role for CrkL and an essential, overlapping role for Crk and CrkL in GBM cell invasion. The results of these cell migration and invasion experiments are consistent with those in our previous report (53).

### Effects of overexpression following knockdown

**Figure 7:**
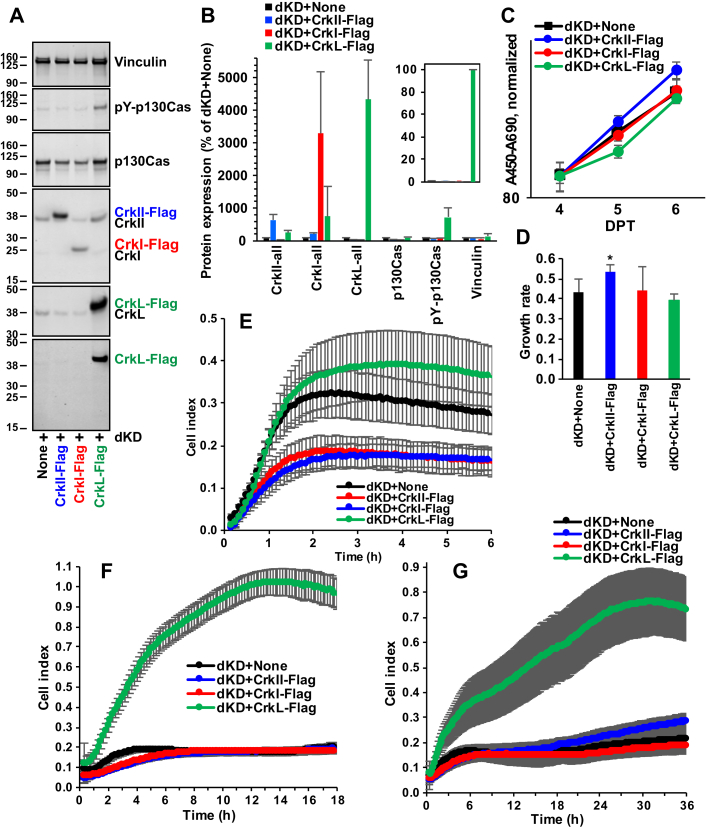
**Effects of overexpression following Crk and CrkL knockdown.** U-118MG cells were first electroporated with Crk siRNA 10 (40 pmol) and CrkL siRNAs 22 (20 pmol) plus 24 (20 pmol) together. At 3 DPT, the cells were harvested, electroporated again with *synRNA* of Flag-tagged CrkII (6 μg), CrkI (6 μg), or CrkL (2 μg), and re-plated for the various assays. The amount of *synRNA* corresponds to approximately 4.4 pmol for CrkL, which produced an effective transfection with *synGFP* (Fig. S1) and in our previous study (52). *A*, at 4 DPT, total cell lysates were obtained for Western blot analyses. Protein levels upon siRNA transfection were compared with those upon electroporation without *synRNA*. Vinculin levels were measured as controls. *B*, protein bands were quantified using the Odyssey system, calculated as percentages of the control (none) signal, and their mean ± SD values are shown. *Inset*, protein bands detected with anti-Flag antibody were quantified, calculated as percentages of the maximal signal (dKD + CrkL-Flag), and their mean ± SD values are shown. The signals from the other three samples were negligible. *C*, proliferation of U-118MG cells electroporated first with siRNAs and then again with *synRNA* was quantitatively measured using WST-1. The A_450_–A_690_ values are presented in the logarithmic scales. *D*, exponential trendlines for the WST-1 assay graphs were drawn and their slopes, the coefficients of *x*, are presented as the rates for exponential cell growth. ∗*p* < 0.05, compared with double knockdown only. *E*–*G*, U-118MG cells electroporated first with siRNAs and then again with *synRNA* were plated onto an E-Plate 16, and two CIM-Plate 16 without and with Matrigel coating for cell adhesion (*E*), migration (*F*), and invasion (*G*) using the xCELLigence system according to the Experimental procedures. Cell index values were obtained from four wells for each sample, and their mean ± SD values are shown. Three independent experiments were carried out, and the results were reproducible. CIM, cell invasion and migration; Crk, CT10 regulator of kinase; CrkL, Crk-like; DPT, days posttransfection; NT1, nontargeting siRNA 1.

We investigated whether the effects of gene knockdown were reversible and rescuable by re-expression of individual proteins. To accomplish this task, we induced Crk/CrkL double knockdown first and then harvested cells at 3 DPT to transfect them with *synRNA* for Flag-tagged CrkII, CrkI, or CrkL. Examination of the protein levels at 4 DPT after the two transfections showed expression of both the endogenous, residual CrkII, CrkI, and CrkL and the overexpressed Flag-tagged counterparts, although Flag-tagged CrkII and CrkI were detected only with anti-Crk and anti-CrkL antibodies (Fig. 7, *A* and *B*). Detection of overexpressed Flag-tagged proteins using an anti-Flag antibody suggested that the level of Flag-tagged CrkL was much higher than Flag-tagged CrkII or CrkI (Fig. 7*B*, inset). Analyses of cell proliferation indicate that overexpression of CrkII moderately increased cell proliferation (Fig. 7, *C* and *D*). On the other hand, cell adhesion to fibronectin was not restored by overexpression of any of these proteins (Fig. 7*E*). The morphological alterations caused by the Crk/CrkL double knockdown were modestly rescued by CrkL overexpression (Fig. S7). Examination of cell migration suggests that only CrkL overexpression restored the migration of cells with Crk/CrkL double knockdown (1.02 ± 0.07 at 14 h, compared with 0.18 ± 0.02 at 14 h for double knockdown only) (Fig. 7*F*). We also examined cell invasion to test whether overexpression of CrkII, CrkI, and CrkL could rescue defects caused by Crk/CrkL double knockdown. Defective cell invasion resulting from Crk/CrkL double knockdown was restored by CrkL overexpression (0.76 ± 0.13 at 30 h, compared with 0.20 ± 0.05 at 30 h for double knockdown only) (Fig. 7*G*). A modest rescue of cell migration and invasion by CrkL overexpression in an independent experiment (data not shown) together with the failure of rescue by CrkII or CrkI overexpression suggests that the defects induced by Crk/CrkL knockdown may be partially irreversible. Nevertheless, the rescue by CrkL overexpression, together with the prominent defects caused by CrkL knockdown, implies that CrkL plays predominant roles in cell migration and invasion in the GBM cell line U-118MG.

### Quantification of Crk and CrkL proteins

**Figure 8:**
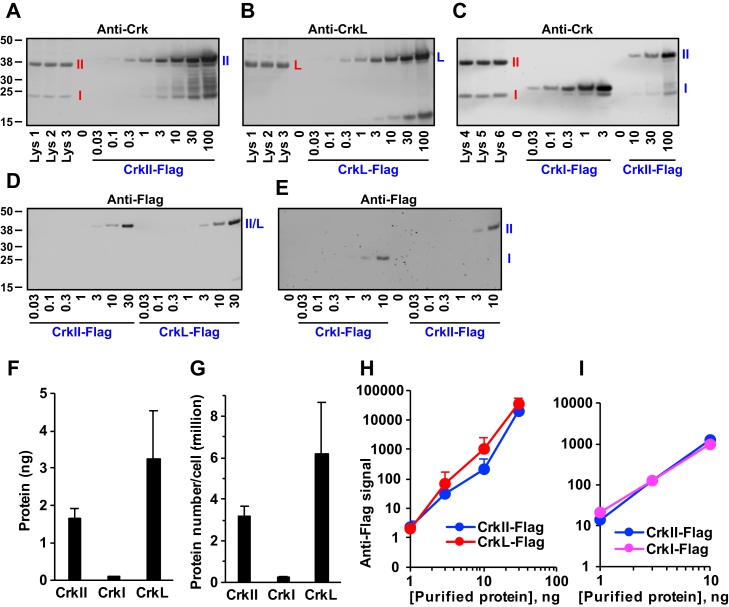
**Quantification of Crk and CrkL proteins using purified proteins.***A*–*C*, Crk and CrkL proteins were detected using anti-Crk (*A* and *C*) or anti-CrkL (*B*) antibodies from three different lysates prepared from U-118MG cells without any transfection (5 μg per lane). The indicated concentrations (ng) of purified proteins (*A*: CrkII-Flag, *B*: CrkL-Flag, and *C*: CrkI-Flag) were loaded together as the standard proteins. Endogenous Crk and CrkL proteins are marked in *red*. Purified proteins are marked in *blue*. *F*, for each Western blot image, protein bands were quantified using the Odyssey system, a standard curve was drawn for the purified protein, and a fourth-order polynomial trendline was obtained using the Microsoft Excel program. The trendline was used to determine the protein concentrations in the cell lysates. These procedures were repeated two more times for the same set of samples, and the mean ± SD values of the three different cell lysates are shown. *G*, U-118MG cells were plated onto 35 mm dishes with similar densities to those for Western blot experiments. One day later, cells were harvested using trypsin-EDTA, counted, and lysed for total protein preparation. Both the cell numbers and the total protein amounts were determined from ten different dishes, and the mean of the total protein per cell was calculated to be 536 pg. This number was used to estimate the cell numbers for the U-118MG cell lysates that were prepared without counting. Protein molecular weights were calculated on http://web.expasy.org/protparam/. Then, the numbers of the protein molecules per cell were calculated. *D* and *E*, the indicated concentrations (ng) of purified Flag-tagged Crk and CrkL proteins were detected using an anti-Flag antibody. *H*–*I*, Protein bands for Flag-tagged Crk and CrkL proteins were quantified using the Odyssey system, and their mean + SD values are shown in logarithmic scales. Crk, CT10 regulator of kinase; CrkL, Crk-like.

Because two different antibodies have been used to detect CrkII and CrkL, the relative abundance of the two proteins in the cells was not determined. To quantify expression levels of Crk and CrkL proteins in mammalian cells, we constructed prokaryotic expression plasmids for CrkII, CrkI, and CrkL with epitope tags, glutathione-S-transferase (GST) and Flag, at N termini and C termini. Then, we purified proteins without the GST tag that still harbor the Flag tag. SDS-PAGE and Coomassie Blue staining of purified proteins exhibited a single band with the expected size and clean background (Fig. S9*A*), suggesting that proteins were pure. Then, we calculated the purity of the proteins by measuring densities of the major bands in comparison with the entire gel lane. The ratios of the major bands to the whole lanes were 98 ± 9% for CrkII, 91 ± 7% for CrkI, and 91 ± 13% for CrkL (Fig. S9*B*), suggesting preparation of highly purified proteins. As shown in Figure 8, *A*–*C*, U-118MG cell lysates were compared with known concentrations of purified proteins, and the protein concentrations in the cell lysates were determined. Figure 8*F* indicates that 1.67 ± 0.25 ng of CrkII and 3.24 ± 1.30 ng of CrkL were present in the 5 μg cell lysate. As CrkI bands were much weaker than CrkII bands in our Western blot experiments, only 0.09 ± 0.01 ng of CrkI was present. Calculation of the protein molecule numbers indicated 3.2 million of CrkII and 6.2 million of CrkL proteins in one U-118MG cell (Fig. 8*G*). On the other hand, the number of CrkI proteins was much lower, with 0.25 million. Comparing the reactivities of the anti-Flag antibody among the purified proteins suggests that the antibody reactivities are comparable among CrkII, CrkI, and CrkL (Fig. 8, *D*, *E*, *H*, and *I*). Together, these results suggest that CrkL is expressed more abundantly than CrkII and CrkI, with CrkI the least abundant protein among the three proteins.

## Discussion

Crk and CrkL are overexpressed in human GBM tissues, and the overexpression contributes to poor prognosis (8, 9, 10, 31). Overexpression of CrkI was reported to increase cell motility and invasion in U87MG cells in a study in which the roles of CrkII and CrkL were not examined (8). On the other hand, two research groups studied the effects of Crk or CrkL knockdown in GBM cell lines. Wang *et al.* (31) established three KMG4 cell lines with stable Crk knockdown. All three Crk knockdown cell lines showed reduced protein levels for CrkII and CrkI. The specificity of the stable knockdown is less clear because the researchers did not examine the effects of shRNAs on CrkL expression. The Crk knockdown partially inhibited the cell adhesion to laminin but not to fibronectin, cell motility assessed by the wound-healing assay, anchorage-dependent cell proliferation, soft agar colony formation, and *in vivo* tumor growth. Lv *et al.* (32) induced CrkL knockdown using CrkL siRNAs in U87 and U251 GBM cell lines. They did not examine the potential effects of the CrkL siRNA on the expression of CrkII and CrkI. CrkL knockdown partially inhibited TGFβ-induced migration and soft agar colony formation of the two GBM cell lines. Although the two knockdown studies demonstrated the important roles of Crk and CrkL in GBM cells, the respective contributions remained unclear.

Crk and CrkL are also elevated in other types of cancer and suggested as therapeutic targets. Despite their crucial overlapping functions in several biological processes, their potential overlapping roles in tumorigenesis have not received much attention. Previously, we used the Cre-loxP recombination to induce single and double knockouts of Crk and CrkL in knockout mouse models and at the cellular level and studied distinct and overlapping functions of Crk and CrkL in tissue development and cellular processes. In this study, we took a gene knockdown approach to induce individual and combined knockdown of Crk and CrkL in human GBM cell lines. Establishing stable cell lines requires a long-term selection *in vitro*, which is susceptible to unrecognized changes in cellular properties with potential changes in the outcome of the study. Short-term induction of gene knockdown using siRNAs is an alternative approach to avoid this problem if the efficiency of knockdown is high. Previously, we achieved a rapid and efficient transfection of fibroblasts with *synRNA* using the Neon electroporation system (52). Here, we used the same system and achieved an efficient transfection of a GBM cell line with siRNAs. Because Crk and CrkL are structurally similar to each other, we carefully examined the cross-activities of siRNAs. In addition to examining the protein levels of CrkII, CrkI, and CrkL as well as control proteins, we compared cellular phenotypes induced by multiple siRNAs. Testing multiple siRNAs together with a close examination and conservative interpretation of the correlation between the knockdown effects and other phenotypes allowed us to tease out nonspecific effects from specific knockdown effects. For example, if two siRNAs exhibited similar knockdown effects in Western blot experiments, but only one siRNA showed additional cellular phenotypes, we interpreted the additional phenotypes as nonspecific effects. By taking this approach, we were able to select more specific and potent siRNAs.

Selected siRNAs for Crk and CrkL were used to induce individual and combined knockdown in U-118MG cells, and a range of cellular phenotypes were examined. Crk knockdown did not affect cell morphology, proliferation, adhesion, and invasion. In contrast, CrkL knockdown caused modest changes in cell morphology, proliferation, adhesion, and invasion. These phenotypic changes were augmented by Crk/CrkL double knockdown, compared with CrkL knockdown. On the other hand, cell migration was delayed by Crk knockdown with slower rates of migration, but the maximal cell migration was comparable to controls. In contrast, CrkL knockdown substantially inhibited cell migration with slower rates of cell migration and reduced the maximal migration. It is unclear why Crk and CrkL affected GBM cell migration differently. One clue is that Crk knockdown did not affect cell morphology or proliferation. Thus, it may take more time, in the absence of Crk, for cells to reorganize cytoskeletal structures in response to changes in the extracellular environment. Cells may eventually catch up and complete cytoskeletal reorganization involved in cell spreading and proliferation. It should be noted that cell morphology and proliferation were analyzed at a single time point each day, whereas real-time measurement of cell migration occurred over a prolonged period. If we had measured only the maximum level of migration, we might have missed delayed cell migration induced by Crk knockdown. This postulation reaffirms the advantage of impedance-based real-time measurement of cell migration, as reported recently (53). However, this interpretation seems to conflict with a lack of inhibitory effects on cell adhesion and invasion by Crk knockdown, both of which were also measured in real time. These results indicate that there may be other mechanisms of Crk function. CrkL appears to be an indispensable component of the cytoskeletal machinery in U-118MG cells. Cells lacking CrkL failed to complete cytoskeletal reorganization upon changes in extracellular environments, leading to partially defective cell spreading, proliferation, and migration.

Our determination of the protein molecule number for CrkII, CrkI, and CrkL provides quantitative insights into the actions of Crk and CrkL. Using purified proteins as the standards, we quantified Crk and CrkL proteins in the cell lysate and protein molecule numbers in a single GBM cell. CrkL is expressed more abundantly than CrkII and CrkI, suggesting that the more abundant expression of CrkL may attribute to the predominant role of CrkL. U-118MG cells may have an intracellular environment that favors CrkL over CrkII and CrkI, leading to a higher overexpression of synthetic CrkL mRNA and a subsequent rescue of the phenotypes.

Although Crk and CrkL appear to act differently during cell migration, Crk/CrkL double knockdown completely blocked migration, suggesting that Crk and CrkL cooperate to play essential overlapping roles in migration of the GBM cell model we tested. More prominent inhibition of cell proliferation, adhesion, and invasion by Crk/CrkL double knockdown than that caused by CrkL knockdown indicates that some of the Crk functions can be revealed only in the absence of CrkL. Because Crk knockdown alone did not reveal contributions of Crk to these cellular functions, our study demonstrates the importance of combining single and double knockdown strategies to fully understand the functions of Crk and CrkL in cancer.

The phosphorylation level of p130Cas correlated well with the protein levels of Crk and CrkL. The substantial decrease in the phospho-p130Cas level with Crk/CrkL double knockdown in GBM cells is consistent with our previous studies with fibroblasts (51, 52) and colorectal and pancreatic cancer cell lines (42). p130Cas is a key binding partner of Crk and CrkL and plays important roles in actin cytoskeleton remodeling, contributing to cell adhesion, spreading, and motility (56). Thus, it will be interesting to investigate whether the Crk/CrkL-p130Cas interaction is critical in the cellular functions mediated by Crk/CrkL and p130Cas.

Because GBM is divided into several subtypes and is highly heterogeneous (3, 57, 58, 59), further *in vitro* and *in vivo* studies using additional GBM cell lines and patient tumor tissues are needed to validate the overlapping functions of Crk and CrkL in GBM. Nevertheless, our study suggests that targeting both Crk and CrkL at the same time may need to be considered to achieve effective therapy. Our study demonstrates that migration of U-118MG cells depends entirely on Crk and CrkL, and that real-time measurement of U-118MG cell migration is a reliable assay to investigate Crk and CrkL activity during development of inhibitors of these proteins. By comparing the migration rates and the maximum levels of migration between control and test groups, we hope to identify drug candidates that inhibit Crk, CrkL, or both.

## Experimental procedures

### Cell culture and transfection

Human GBM cell lines, including U-118MG, were purchased from American Type Culture Collection (ATCC), cultured in Dulbecco's modified Eagle's medium (DMEM) (ATCC) supplemented with 5% fetal bovine serum (FBS) (HyClone) and 1% penicillin/streptomycin (Gibco) at 37 °C under 5% CO_2_ and cryopreserved for future experiments according to the ATCC's instructions. Before we started our planned experiments, the cell lines were authenticated using short tandem repeat profiling through the ATCC. For the gene knockdown study, human *CRK* siRNAs 7 (J-010503-07), 8 (J-010503-08), 9 (J-010503-09), and 10 (J-010503-10) and human *CRKL* siRNAs 6 (J-012023-06), 7 (J-012023-07), 8 (J-012023-08), and 9 (J-012023-09), as well as nontargeting siRNA 1 (NT1, D-001818-01), were purchased from GE Dharmacon. Human *CRKL* siRNAs 22 (s3522), 23 (s3523), and 24 (s3524) were purchased from Ambion. The *synRNAs* were synthesized *in vitro* using the Megascript T7 reaction (Invitrogen) and the EPAP poly-A tailing system (Ambion), as described previously (52). For the transfection of GBM cells with siRNAs or *synRNA*, cells were harvested and counted, and 1 × 10^5^ cells were gently mixed with the indicated amounts (μg in 10 μl cell suspension) of RNAs. Then, the U-118MG cells were placed in a 10 μl Neon tip and electroporated with three pulses at 1350 V for 10 ms using the Neon transfection system (Life Technologies). The manufacturer's instructions for the Neon system recommend using 0.5 to 3 μg DNA or 10 to 200 nM siRNA for electroporating cells to plate in a six-well with 2 ml of culture medium. We achieved efficient transfection of U-118MG cells by using 2 μg *synGFP*, as shown in Figure S1. Because the volume for electroporation is limited, we first used 20 nM to test siRNAs, which corresponds to 40 pmol. However, because the seeding density of cells and the amount of culture medium changed depending on the type of analysis, we used moles rather than molar concentrations to indicate more accurately how much siRNA was used for each 10 μl electroporation. Where indicated, cells were electroporated with 2 to 6 μg *synRNA* of Flag-tagged CrkII, CrkI, or CrkL. The 2 μg *synRNA* corresponds to approximately 4.4 pmol for CrkII and CrkL and 5.7 pmol for CrkI, which produced effective transfection of fibroblasts in our previous study (52). Transfected cells for each sample were pooled in the culture medium without antibiotics and seeded onto 48-well plates for cell proliferation analysis using WST-1 (Roche) (9000 cells/well), 8-well culture slides (BD) for immunocytochemistry (9000 cells/well), and 35 mm dishes for protein expression analysis using Western blot analysis (100,000 cells/dish). Where indicated, transfected cells were plated onto 100 mm dishes and cultured for 3 days before they were harvested for the second transfection and subsequent analyses, including real-time measurement of cell adhesion (10,000 cells/well), migration (100,000 cells/well), and invasion (100,000 cells/well). Images of live cells were taken using the EVOS system with PL FL 10x LWD PH (ThermoFisher Scientific) before cell lysates were prepared from 35 mm dishes.

### Western blot analysis

Both preparations of GBM cell lysates and Western blot analyses using the Odyssey infrared imaging system (Li-Cor Biosciences) were described previously (60). Protein bands from at least three Western experiments were quantified using the Odyssey system, and their mean ± SD values are shown in the graph. The following antibodies were used: anti-Crk (610035) and anti-p130Cas (610272) antibodies are from BD Biosciences; anti-CrkL (sc-319) antibody is from Santa Cruz Biotechnology Inc; anti-alpha-tubulin (T9026) antibody is from Sigma; antibodies against phospho-p130Cas (Tyr410; 4011) and vinculin (4970) are from Cell Signaling Technology. Two prestained protein ladders, Chameleon Duo (Li-Cor Biosciences) and SeeBlue Plus2 (ThermoFisher Scientific) were used to determine the size of proteins.

### Cell proliferation assay using WST-1

Cell proliferation was measured using cleavage of the tetrazolium salt WST-1 (Roche Applied Science) into a water-soluble formazan by cellular enzymes, as described previously (52). Cells were seeded in quadruplicate at 9 × 10^3^ cells/well onto 48-well plates after transfection. For the cell proliferation measurement, cells were incubated with 200 μl/well of fresh culture medium plus 20 μl/well of WST-1 stock solution provided by the manufacturer for 2 h in a CO_2_ incubator. As a background control, WST-1 was added to the medium without cells. At the end of the incubation, the conditioned medium was mixed for 1 min on a shaker to make the medium homogenous, and absorbances at both 450 and 690 nm were read using the Infinite M200 PRO NanoQuant microplate reader (Tecan) for measurement and reference wavelengths, respectively. The reference value was subtracted from the measurement value, and the A_450–690_ value was multiplied by 1000 for easy calculation and presentation purposes. Where indicated, results were normalized to the percentages of the value obtained at the first measurement for the sample (1 DPT, or 4 DPT if cells were harvested at 3 DPT). Results were plotted on a graph using Microsoft Excel with the *x*-axis for DPT and the *y*-axis for A_450–690_ in the logarithmic scale. An exponential trendline was obtained by using the Microsoft Excel program, and the slope of the trendline, which is the coefficient of *x*, is presented as the rate for exponential cell growth.

### Impedance-based real-time cell assays

Cell adhesion to fibronectin-coated dishes was measured in real-time using the xCELLigence Real-Time Cell Analyzer (Agilent) according to the manufacturer's instructions. Briefly, E-plate 16 was coated with 20 μl/well of 10 μg/ml fibronectin. Next, 50 μl/well of DMEM containing 0.5% FBS was added to the E-Plate 16 for the baseline measurement. Then, 1 × 10^4^ cells in 100 μl of DMEM containing 0.5% FBS were added to each well of the E-Plate 16, and impedance was measured every 5 min for the indicated time. Cell migration was also measured in real time using xCELLigence, as described previously (53). Briefly, the upper and lower chambers of a cell invasion and migration (CIM) plate with 16 wells (CIM-Plate 16) were filled with 50 μl of low-serum (0.5% FBS) medium and 160 μl of high-serum (10% FBS) medium, respectively. After the upper and lower chambers were assembled as instructed by the manufacturer, the CIM-Plate was placed in a CO_2_ incubator for 30 min to establish the serum gradient. Then, the baseline was measured using xCELLigence located inside a CO_2_ incubator. After 1 × 10^5^ cells in 100 μl of low-serum medium were added to each well of the upper chamber, the CIM-Plate was kept at room temperature for 30 min to allow the cells to settle down at the bottom. Then, the CIM-Plate was transferred to a cradle of xCELLigence, and changes in the cell impedance were measured every 10 min for 24 h. Cell index data were exported to Microsoft Excel for processing and graphical presentation in a time-dependent manner. The time of starting to measure the impedance after the addition of cells to the CIM-Plate, not the time when the baseline was measured, was set as the start time for plotting graphs. For the cell invasion assay, the upper chamber of the CIM-Plate was coated with 2 μg/well of Matrigel (Corning), and the rest of the procedures were the same as the cell migration assay.

### Immunocytochemistry and cytometric analyses

Immunostaining of GBM cells cultured on 8-well culture slides was carried out as previously reported (60) with some modifications. High magnification images of immunostained cells were captured using a Leica DM6B microscope equipped with Leica HC FL Fluotar 10x/0.32 PH1, Hamamatsu Orca Flash 4.0 V3 monochrome, and Leica DFC450 color cameras. Quantitative assessments of transfection efficiency and cell morphology were performed by the fluorescence imaging-based method using the LAS X program. For quantification of the cytoplasmic and nuclear areas, phalloidin-TRITC and DAPI images of cells were taken throughout the entire area of the well. Areas and numbers of DAPI-positive objects were calculated to obtain the average size of the nucleus for each field of view. Then, the total cytoplasmic area with phalloidin signals was divided by the number of DAPI-positive objects to obtain the average size of the cytoplasm for each field of view. The values from all the fields of view were combined to calculate the overall sizes of the nucleus and cytoplasm.

### Flow cytometry

Single-cell suspensions were fixed and permeabilized using BD Cytofix/Cytoperm and stained with mouse anti-Crk (BD Biosciences, 610035) and rabbit anti-CrkL (Santa Cruz Biotechnology, sc-319) antibodies, followed by APC-conjugated anti-mouse IgG (BD Biosciences, 550826) and BV421-conjugated anti-rabbit IgG (BD Biosciences, 565014). Cells were analyzed by flow cytometry using the On-chip Sort (On-chip Biotechnologies).

### Protein purification from bacterial cells

Mouse cDNA sequences of CrkII, CrkI, and CrkL with the Flag tag were subcloned into the pGEX-4T-3 vector (GE Healthcare). BL21(DE3) competent cells (New England Biolabs) were transformed with the plasmids, and expression of the GST-fusion proteins was induced by adding isopropyl β-D-1-thiogalactopyranoside to the bacterial culture. Bacterial cells were harvested, resuspended in phosphate buffered saline, and lysed mechanically with a sonic dismembrator (Fisher Scientific). GST-fusion proteins were isolated with glutathione agarose (Pierce) and treated with thrombin (GE Healthcare) or reduced glutathione (Fisher Scientific) to collect the whole protein or the protein without the GST tag. Concentrations of purified proteins were determined by using the Bio-Rad protein assay kit. One microgram of each purified protein was loaded on an Invitrogen NuPAGE 4 to 12% Bis-Tris gel (Thermo Fisher) and separated by SDS-PAGE. For Coomassie Blue staining, the gel was stained with the GelCode Blue stain reagent (Thermo Scientific) according to the manufacturer's instructions. The gel was scanned, and its image file was imported to the Odyssey infrared imaging system (Li-Cor Biosciences). Densities of the major bands and the entire gel lanes (from 160 to 15 kDa) were quantified, and the ratios of the major bands to the entire lanes were calculated to determine the purity of the bands.

### Statistical analysis

All quantitative data are presented as mean ± SD. Statistical analyses of data were carried out using unpaired two-tailed Student's *t* test for comparison between two experimental groups. Differences were considered to be significant when probability (*p*) values were <0.05.

## Data availability

The data supporting the findings of this study are contained in the article and its supporting information. The data that are described but not shown in the article will be shared upon request by the corresponding author (Taeju Park, Children's Mercy Kansas City, tjpark@cmh.edu).

## Conflict of interest

The authors declare that they have no conflicts of interest with the contents of this article.
