# Supplementary material for: Quantitative assessment of glioblastoma phenotypes in vitro establishes cell migration as a robust readout of Crk and CrkL activity
Source: J Biol Chem. 2021 Feb 6;296:100390. doi: 10.1016/j.jbc.2021.100390 (PMC7961105; doi:10.1016/j.jbc.2021.100390)
Supplement: Figures S1 to S9 [file mmc1.pdf]

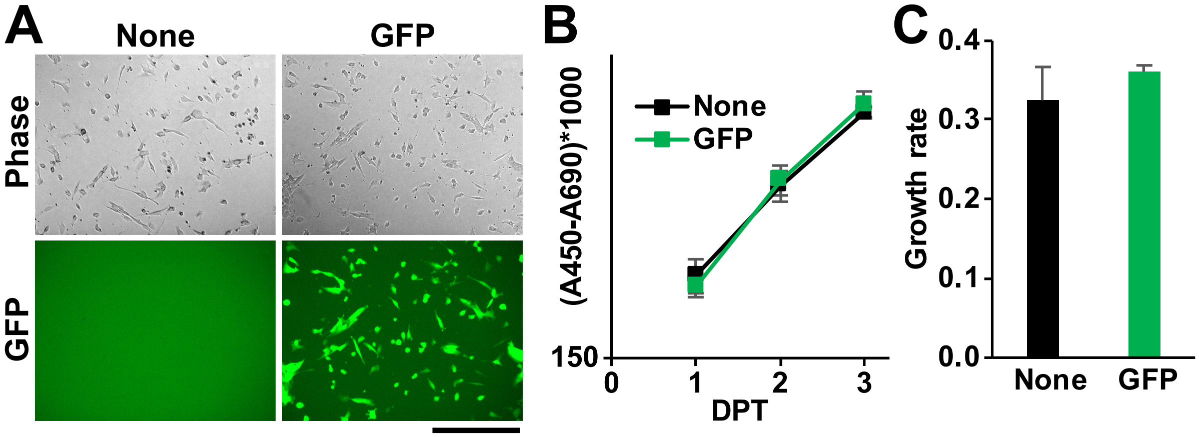

Suppl. Figure 1. Electroporation of U-118MG with synGFP. A. After U-118MG cells were electroporated with *synGFP* (2 µg in 10 µL cell suspension), both phase-contrast and GFP images of cells were taken at 1 day post-transfection (DPT). Scale bar: 400 µm. **B.** Proliferation of U-118MG cells after *synGFP* transfection was quantitatively measured using WST-1, and the OD450 – OD690 values are presented in the logarithmic scales. **C.** Exponential trendlines for the WST-1 assay graphs were drawn, and their slopes, the coefficients of χ, are presented as the rates for exponential cell growth.

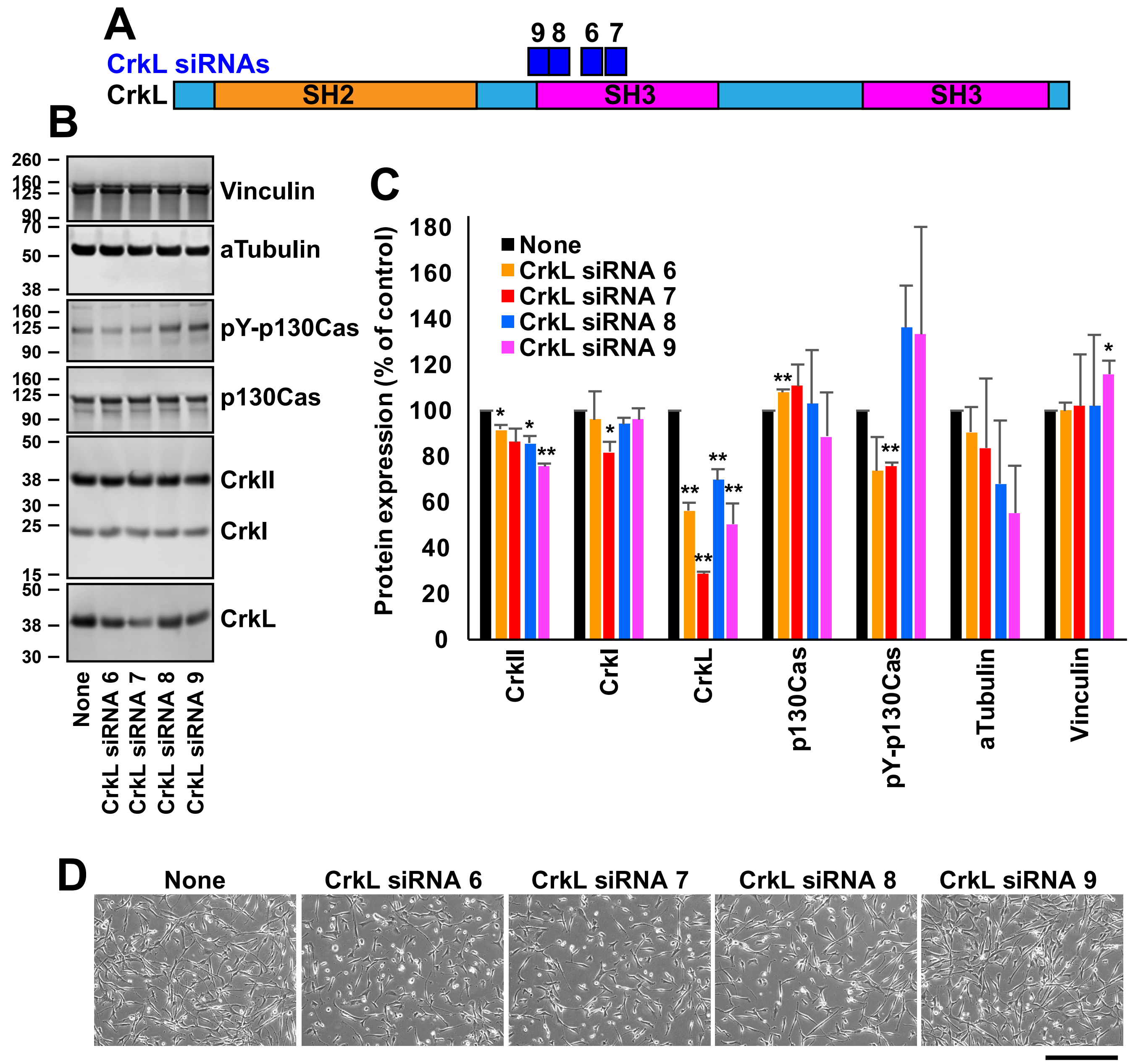

Suppl. Figure 2. Electroporation of U-118MG cells with CrkL siRNAs. A. A schematic diagram of the target areas of CrkL siRNAs. **B.** U-118MG cells were electroporated with CrkL siRNAs (40 pmol in 10 µL cell suspension), and total cell lysates were prepared at 3 DPT for Western blot analyses. Protein levels upon siRNA transfection were compared with those upon electroporation without siRNAs. Alpha-tubulin and vinculin levels were measured as controls. **C.** Protein bands were quantified using the Odyssey system, and their mean ± SD values are shown. *$p < 0.05$, **$p < 0.01$, compared with the control (none). **D.** Phase-contrast images of live cells were taken at 3 DPT using the EVOS system. Representative images are shown. Scale bar: 400 µm.

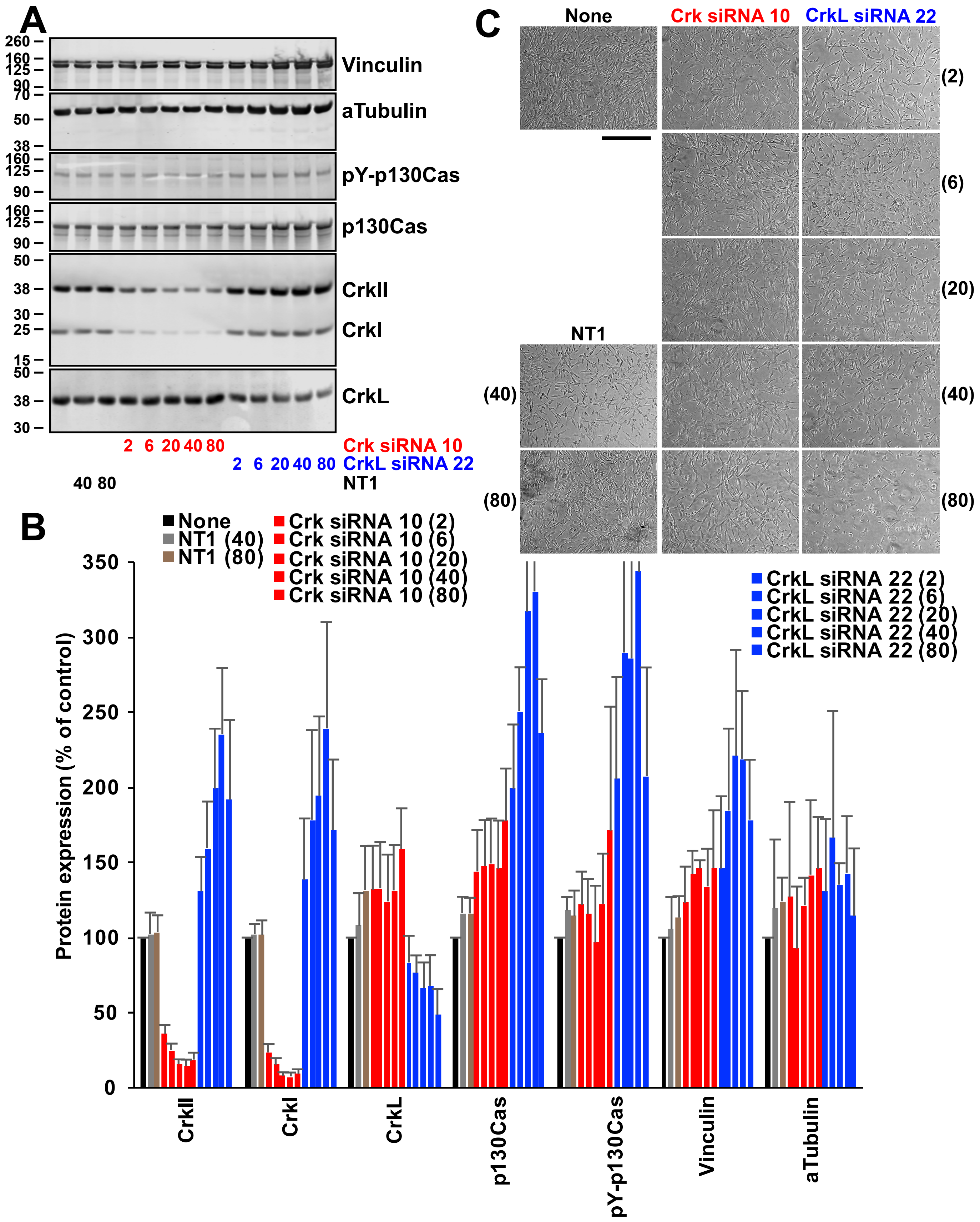

Suppl. Figure 3. Concentration-dependent knockdown by Crk and CrkL siRNAs. A. U-118MG cells were electroporated with 2-80 pmol of Crk siRNA 10 and CrkL siRNA 22, and total cell lysates were prepared at 3 DPT for Western blot analyses. We used moles rather than molar concentrations to indicate accurately how much siRNA was used for each electroporation, as described in the Experimental Procedures. Protein levels upon siRNA transfection were compared with those upon electroporation without siRNAs (none) or with NT1. Alpha-tubulin and vinculin levels were measured as controls. **B.** Protein bands were quantified using the Odyssey system, and their mean ± SD values are shown. **C.** Phase-contrast images of live cells were taken using the EVOS system at 3 DPT. Representative images are shown. Scale bar: 400 μm.

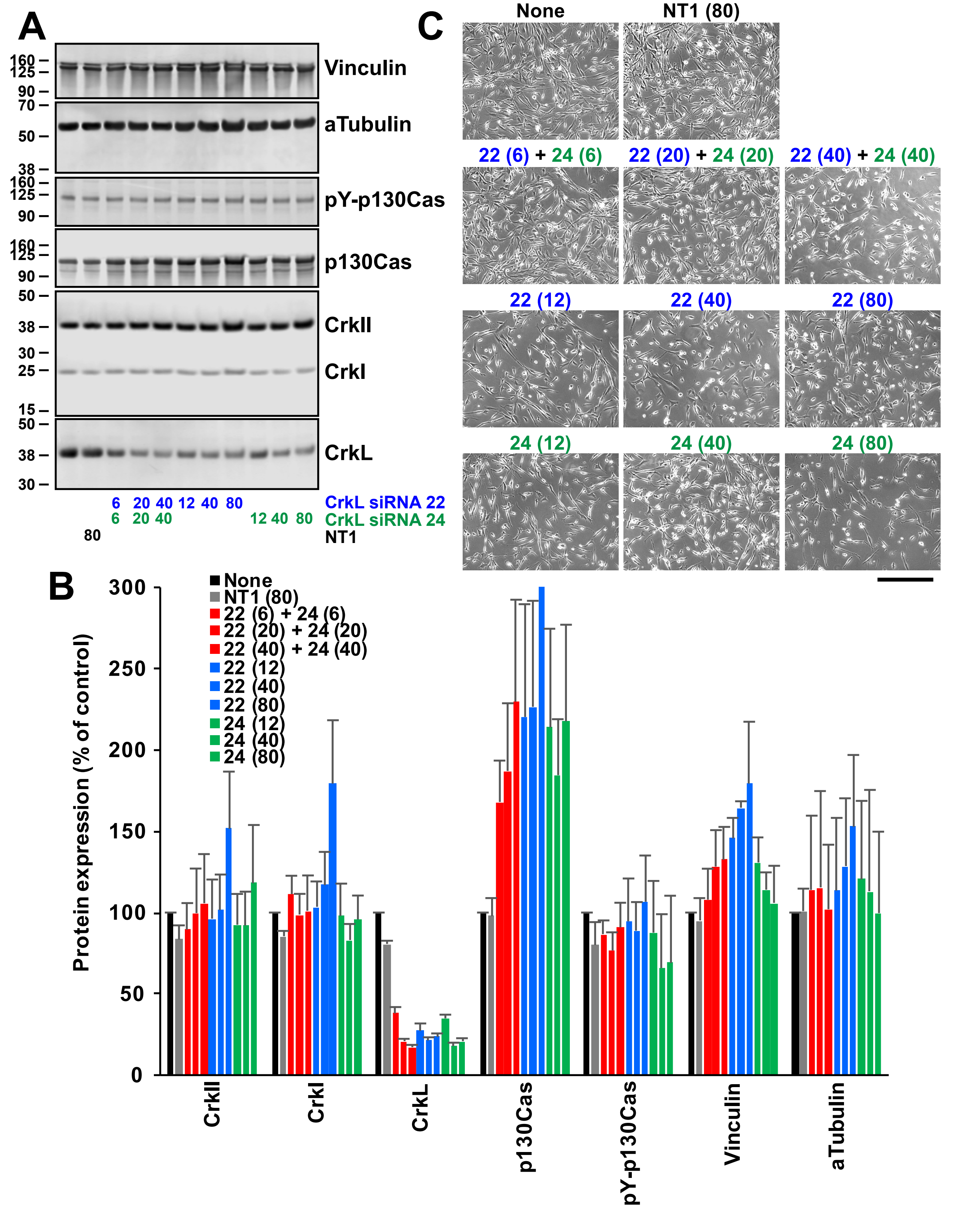

Suppl. Figure 4. Electroporation of U-118MG cells with CrkL siRNAs 22 and 24. A. U-118MG cells were electroporated with different amounts (indicated as numbers in the pmol unit in parentheses) of CrkL siRNAs 22 and 24, and total cell lysates were prepared at 4 DPT for Western blot analyses. Protein levels upon siRNA transfection were compared with those upon electroporation without siRNAs (none) or with NT1. Alpha-tubulin and vinculin levels were measured as controls. **B.** Protein bands were quantified using the Odyssey system, and their mean ± SD values are shown. **C.** Phase-contrast images of live cells were taken using the EVOS system at 4 DPT. Representative images are shown. Scale bar: 400 μm.

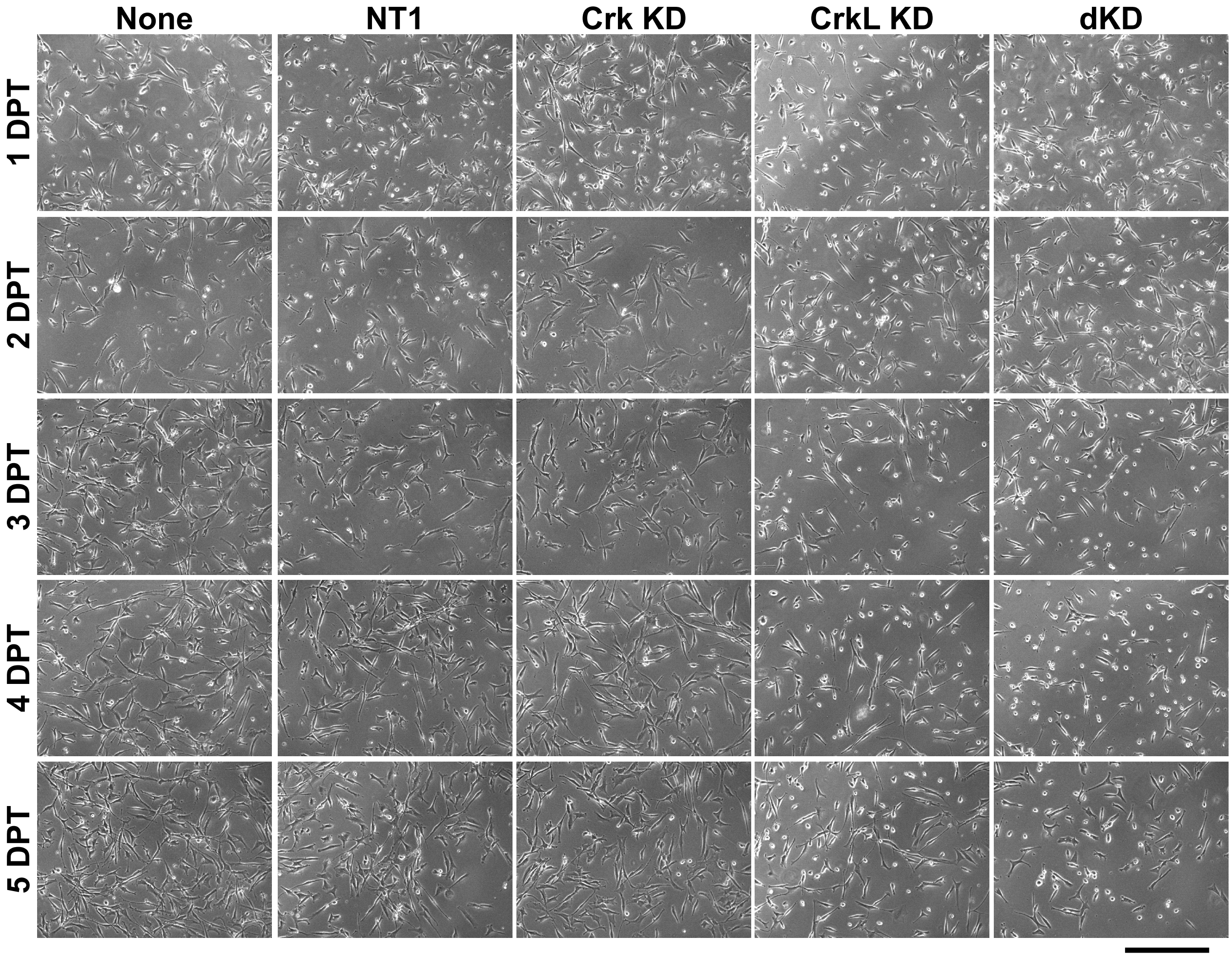

Suppl. Figure 5. Effects of Crk and CrkL knockdown on cell morphology. U-118MG cells were electroporated with NT1 (80 pmol), Crk siRNA 10 (40 pmol), CrkL siRNAs 22 (20 pmol) plus 24 (20 pmol), or with Crk and CrkL siRNAs together, and phase-contrast images of live cells were taken at the indicated DPT using the EVOS system. Representative images are shown. Scale bar: 400 µm. Two independent experiments were carried out, and the results were reproducible.

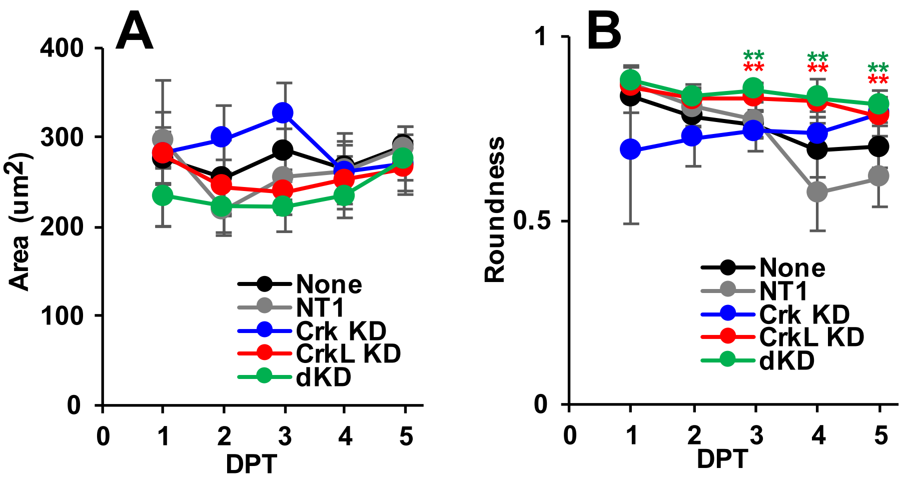

Suppl. Figure 6. Effects of Crk and CrkL knockdown on nuclear morphology. U-118MG cells were electroporated with NT1 (80 pmol), Crk siRNA 10 (40 pmol), CrkL siRNAs 22 (20 pmol) plus 24 (20 pmol), or with Crk and CrkL siRNAs together, and the cells were fixed at the indicated DPT. The fixed cells were stained with DAPI to visualize the nucleus. Nuclear areas (**A**) and roundness (**B**) of DAPI-stained objects were calculated according to the Experimental Procedures, and their mean ± SD values are shown. **$p < 0.01$, compared with both none and NT1 at the same DPT. Two independent experiments were carried out and the results were reproducible.

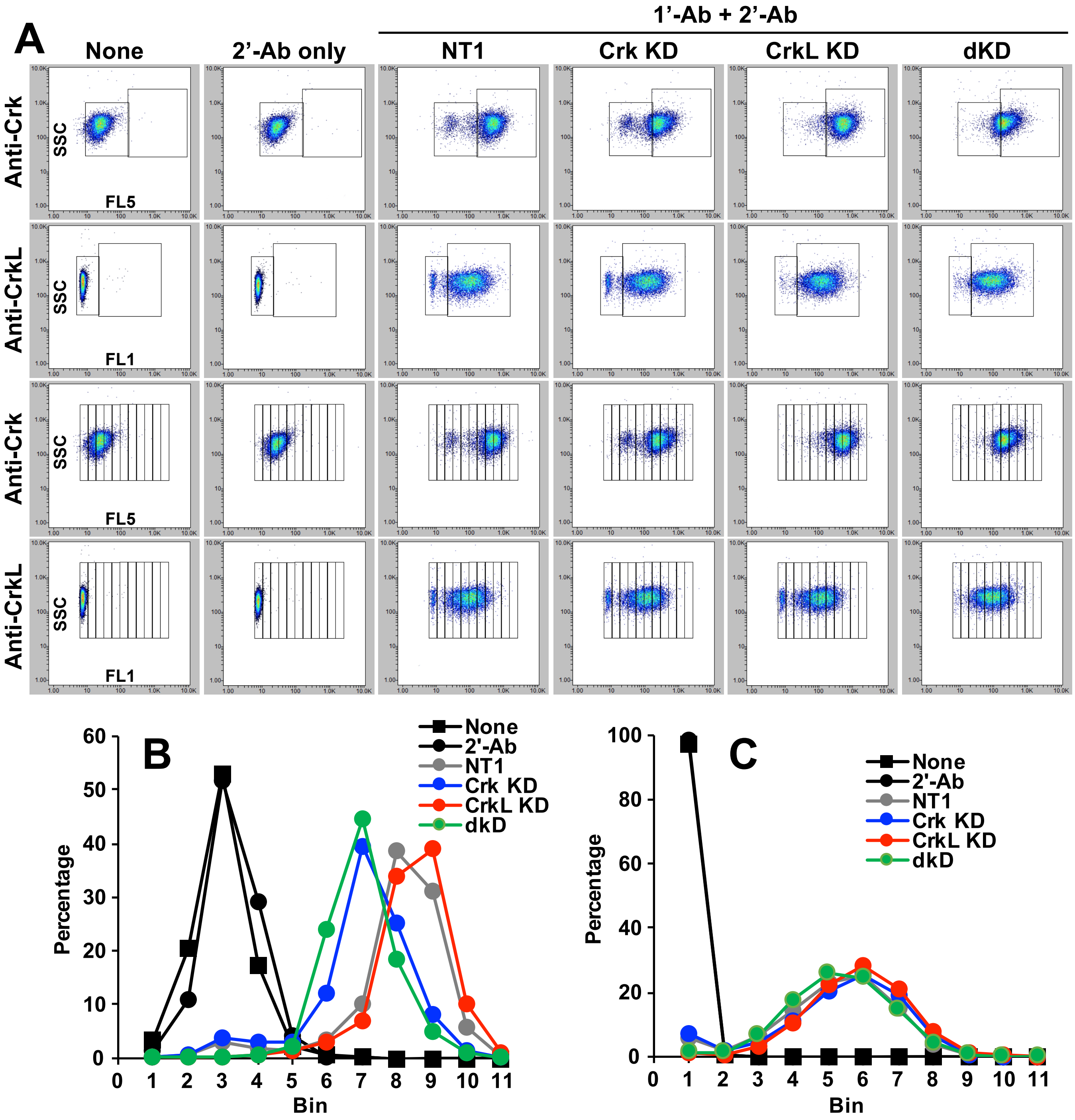

Suppl. Figure 7. Expression of Crk and CrkL after gene knockdown. A. U-118MG cells were electroporated with NT1 (80 pmol), Crk siRNA 10 (40 pmol), CrkL siRNAs 22 (20 pmol) plus 24 (20 pmol), or with Crk and CrkL siRNAs together, and the cells were harvested, fixed, and permeabilized at 4 DPT. The fixed cells were stained for Crk and CrkL according to the Experimental Procedures., and the intracellular staining of Crk and CrkL was determined by flow cytometry. Results are representative of three independent experiments. **B.** The fluorescence signal from anti-Crk antibody was divided into 11 bins depending on the signal strength and the event numbers were plotted for each bin. **C.** The fluorescence signal from anti-CrkL antibody was divided into 11 bins depending on the signal strength and the event numbers were plotted for each bin.

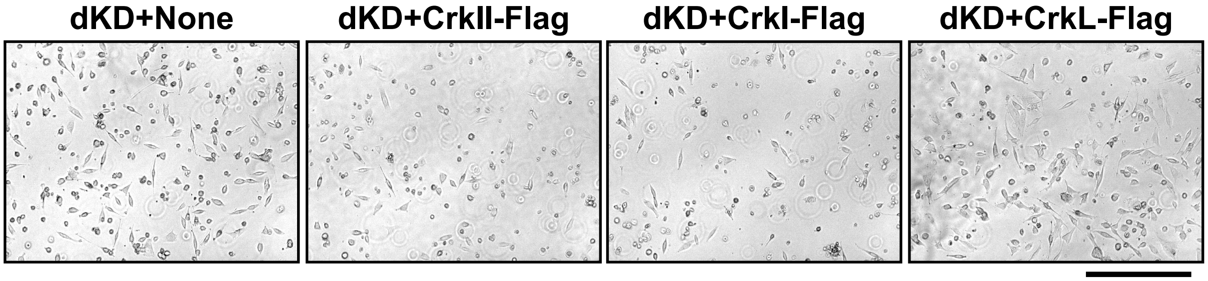

Suppl. Figure 8. Effects of overexpression following Crk and CrkL knockdown on cell morphology. U-118MG cells were first electroporated with Crk siRNA 10 (40 pmol) and CrkL siRNAs 22 (20 pmol) plus 24 (20 pmol) together. At 3 DPT, the cells were harvested, electroporated again with *synRNA* of Flag-tagged CrkII (6 µg), CrkI (6 µg), or CrkL (2 µg), and re-plated. Phase-contrast images of live cells were taken at 4 DPT using the EVOS system. Representative images are shown. Scale bar: 400 µm.

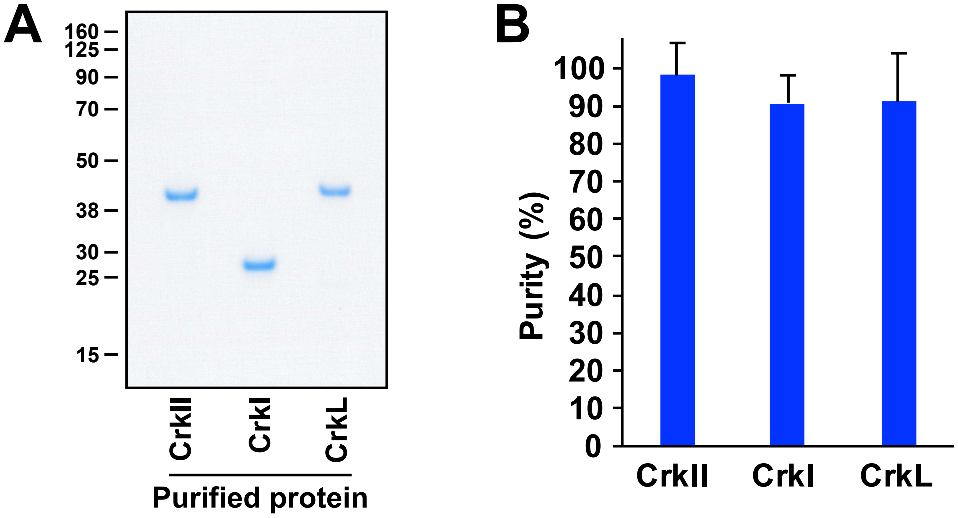

Suppl. Figure 9. Purification of recombinant CrkII, CrkI, and CrkL proteins. A. One microgram of the purified protein without the GST tag was analyzed by SDS-PAGE, and proteins in the gel were stained with GelCode Blue. The gel was scanned, and a representative image is shown. Four independent experiments were carried out, and the results were reproducible. **B.** The gel image was imported to the Odyssey imaging system, and densities of major bands and the entire gel lanes were measured. Ratios of the major bands to the entire lanes were calculated, and their mean ± SD values from four independent experiments are shown.
